# Nodularin Exposure Induces SOD1 Phosphorylation and Disrupts SOD1 Co-localization with Actin Filaments

**DOI:** 10.3390/toxins4121482

**Published:** 2012-12-14

**Authors:** Linda V. Hjørnevik, Lise Fismen, Fiona M. Young, Therese Solstad, Kari E. Fladmark

**Affiliations:** 1 Department of Molecular Biology, University of Bergen, Bergen N-5020, Norway; E-Mails: linda.hjornevik@mbi.uib.no (L.V.H.); lise.fismen@med.uib.no (L.F.); 2 Division of Environmental and Applied Biology, School of Life Sciences, University of Dundee, Dundee DD1 4HN, Scotland, UK; E-Mail: fiona.callum@yahoo.co.uk; 3 Proteomics Unit at University of Bergen, Department of Biomedicine, University of Bergen, Bergen N-5020, Norway; E-Mail: therese.saunders@legemiddelverket.no

**Keywords:** nodularin, apoptosis, SOD1, phosphorylation, actin, ROS

## Abstract

Apoptotic cell death is induced in primary hepatocytes by the Ser/Thr protein phosphatase inhibiting cyanobacterial toxin nodularin after only minutes of exposure. Nodularin-induced apoptosis involves a rapid development of reactive oxygen species (ROS), which can be delayed by the Ca^2+^/calmodulin protein kinase II inhibitor KN93. This apoptosis model provides us with a unique population of highly synchronized dying cells, making it possible to identify low abundant phosphoproteins participating in apoptosis signaling. Here, we show that nodularin induces phosphorylation and possibly also cysteine oxidation of the antioxidant Cu,Zn superoxide dismutase (SOD1), without altering enzymatic SOD1 activity. The observed post-translational modifications of SOD1 could be regulated by Ca^2+^/calmodulin protein kinase II. In untreated hepatocytes, a high concentration of SOD1 was found in the sub-membranous area, co-localized with the cortical actin cytoskeleton. In the early phase of nodularin exposure, SOD1 was found in high concentration in evenly distributed apoptotic buds. Nodularin induced a rapid reorganization of the actin cytoskeleton and, at the time of polarized budding, SOD1 and actin filaments no longer co-localized.

## 1. Introduction

The cyanobacterial-produced hepatotoxins, nodularin and microcystin, are potent inhibitors of the general Ser/Thr phosphatases (PP1 and PP2A). Exposure to these toxins induces apoptotic cell death in isolated hepatocytes after only minutes of exposure [1,2]. The apoptotic cell death is preceded by hyperphosphorylation of a number of different proteins [3,4]. 

Phosphorylated signaling proteins are often present in low amounts in the cells, making identification work difficult. In our rapid apoptosis model, where hepatocytes become apoptotic in minutes, we achieve a unique amount of synchronized cells, thus increasing the possibility to identify low amount phosphoproteins that are involved in pro- or anti-apoptotic cell signaling.

Hitherto, most known phosphorylated target proteins in nodularin and microcystin-exposed apoptotic hepatocytes are cytoskeletal, or proteins known to associate with the cytoskeleton, e.g., plectin [5], keratin 8/18 [6], myosin light chain [1], HSP27 and tau [7,8]. Most likely, these phosphorylation events are coupled to the dramatic cytoskeletal reorganization occurring after phosphatase inhibitor exposure, starting with loss of cell-cell contacts [9], followed by disruption of intermediate filaments [10] and contraction and aggregation of actin microfilaments [11]. These cytoskeletal changes are reflected in the apoptotic morphology of the cell in which microvilli are lost, followed by the appearance of evenly distributed apoptotic buds, which eventually gather at one pole [4].

Our aim is to identify phosphoproteins that are essential for the early regulation of nodularin-induced apoptotic cell death. In the present study, we identified the cytosolic antioxidant Cu,Zn superoxide dismutase (SOD1) as one of these phosphorylated target proteins. SOD1 is a prime defender against oxidative stress [12], but also a regulatory protein with possible implications for a range of sub-cellular localized processes, including cytoskeletal rearrangement [13]. SOD1 is essential for the preservation of the cytoskeleton, thus dysregulation in SOD1 results in cytoskeletal rearrangements [14,15]. We found that the initial co-localization of SOD1 and actin in hepatocytes was disrupted during nodularin exposure and that a high content of SOD1 was found in the apoptotic buds. This may suggest that the nodularin-induced SOD1 phosphorylation has a role in cytoskeletal reorganization by regulating the redox state at specific sub-cellular locations. 

## 2. Results and Discussion

### 2.1. SOD1 Is Phosphorylated in the Early Phase of Nodularin-Induced Apoptosis

**Figure 1:**
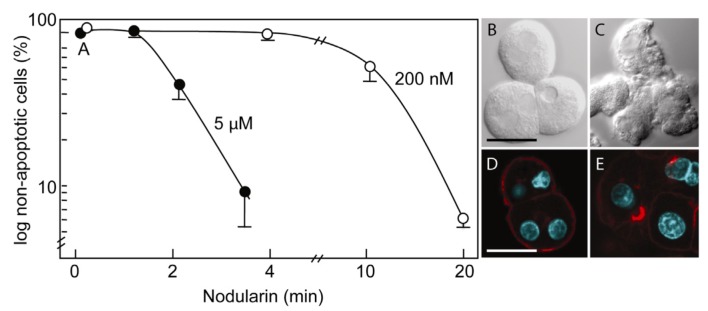
Nodularin-induced apoptosis in primary hepatocytes. Primary hepatocytes in suspension were exposed to 5 µM (●) or 200 nM (○) nodularin (**A**). The unexposed hepatocytes appeared round with a smooth cell surface (**B**), whilst nodularin-exposed (5 µM) hepatocytes showed profound apoptotic-related budding (**C**). Control cells also had and with a cortical actin cytoskeleton (**D**) which was reorganized after nodularin exposure (**E**). Cell death was determined based on the appearance of apoptotic-related morphology. Values are the mean +/− S.E.M. of 3–5 separate experiments. Actin filaments were detected using rhodamin-conjugated phalloidin. Nuclei are stained with DAPI. Bars, 20 μm.

Primary hepatocytes exposed to 5 µM nodularin developed apoptosis, involving membrane budding (Figure 1C) and actin reorganization (Figure 1E), in only 2–4 min after toxin addition (Figure 1). Exposure to 200 nM gave similar morphological changes after 10–20 min of incubation (Figure 1A) [2]. 

**Figure 2:**
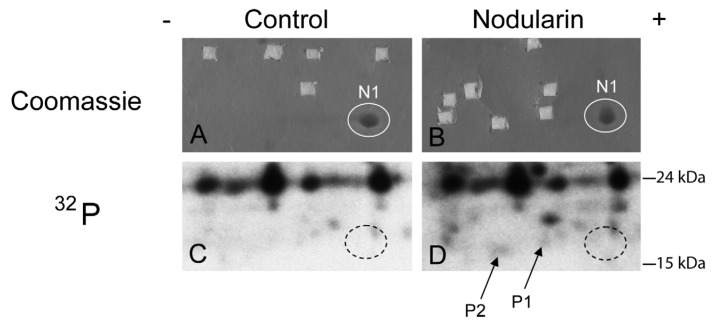
SOD1 is phosphorylated in nodularin-treated apoptotic hepatocytes. Primary hepatocytes in suspension culture were pre-labeled for 35 min with ^32^Pi and exposed to nodularin (200 nM) for 10 min. Hepatocyte extract was fractionated and the cytosolic fraction (2.5 mg protein) was separated by two-dimensional gel electrophoresis (pI 5.3–6.5, 13 cm). Spots were identified using MALDI MS or MS/MS. See Experimental Section for further details. The figure shows Coomassie-stained gels (**A** and **B**) and their corresponding autoradiographs (**C** and **D**). Only non-phosphorylated SOD1 (spot N1) was detectable with Coomassie staining. Spots were therefore cut from the Coomassie-stained gels using the autoradiographs as guidance. In nodularin-treated hepatocytes SOD1 was phosphorylated and appeared as two novel spots with an acidic shift (spot P1 and P2). N1, P1 and P2 were identified as SOD1.

We have previously shown that both nodularin and microcystin induce phosphorylation of a number of proteins before any morphological signs of apoptosis can be observed [1]. These early phosphorylated proteins are of interest as they most possibly are key players in apoptosis development or protection. Hitherto, we have just been able to identify one of these, namely acyl-CoA binding protein [2], using proteomics-based techniques. The challenge is due to the low level amount of these phoshoproteins, as they cannot be visualized with high sensitivity protein staining (silver or Sypro Ruby). To be able to identify early phosphorylation events, we exposed hepatocytes to 200 nM nodularin for 20 min. To increase the amount of phosphoproteins loaded on the gel, the cell lysates were sub-fractionated. Phosphoproteins were visualized by autoradiography after pre-labeling hepatocytes with radioactive phosphate (Figure 2C,D), and the autoradiographs were used as guidance to cut out corresponding unstained spots from the gels (Figure 2A,B). 

**Figure 3:**
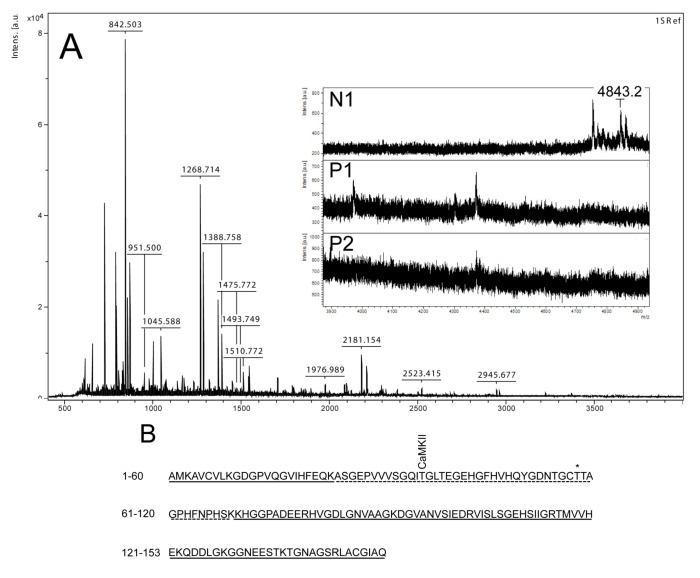
Mass spectrometry analysis of spot N1, P1 and P2. (**A**) MALDI-MS spectrum of tryptic peptides obtained from P1 spot shown in Figure 2. N1, P1, and P2 were all identified as SOD1. Mass spectra were similar for all three spots (N1, P1 and P2), except that the long tryptic peptide (4843.2 *m*/*z*), corresponding to aa 24–69, was only observed in N1 (insert of **A**). Sequence covered by all three spots is underlined in (**B**). Dotted line refers to sequence covered only in N1 and represents the long tryptic peptide aa 24–69. This peptide has several possible Ser/Thr phosphorylation sites including a consensus sequence site for CaMKII and also a previously suggested phosphorylation site (asterisk) (Thr58) [16].

By using the narrow ranged strips (pI 5.3–6.5) along with maximal protein loading, we were able to identify several phosphorylated proteins by mass spectrometry. Two of the phosphorylated spots (P1 and P2) that increased in intensity after nodularin exposure were identified as SOD1 (Figure 2D and Figure 3A). Neither of these was evident by protein staining. On the other hand, a more basic spot (N1) was visualized by Coomassie, both in control and in nodularin-exposed hepatocytes (Figure 2A,B). N1 was shown to be a non-phosphorylated form of SOD1 (Figure 2C,D and Figure 3A insert). The acidic shifts of P1 and P2 from the parental N1 spot, together with their radioactive phosphate labeling, supported that P1 and P2 were phosphorylated forms of N1. In our effort to identify the specific phosphorylation site(s) of SOD1, we did not succeed. One explanation could be that the phosphorylation site(s) might be located in the long tryptic peptide (4843.2 *m*/*z*) with low intensity extending from amino acid position 24 to 69 (Figure 3B). This tryptic peptide was absent in the P1 and P2 spectra (Figure 3A insert), indicating that it may have been post-translationally modified in the nodularin-exposed cells.

**Figure 4:**
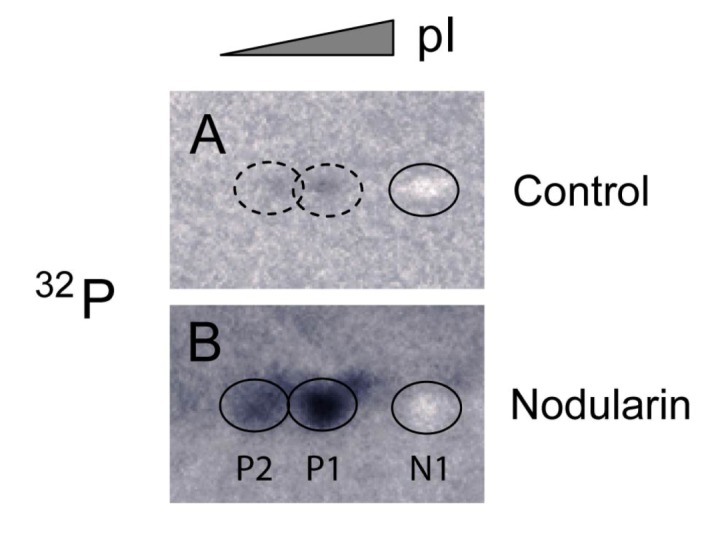
Immunoprecipitation of SOD1 from ^32^P-labeled apoptotic hepatocytes. Hepatocytes were pre-labeled with ^32^Pi and exposed to 5 µM nodularin for 2 min. Total cell extract was immunoprecipitated with anti-SOD1 antibody and separated by two-dimensional gel electrophoresis (pI 4–7, 7 cm). See Experimental Section for further details. The figure shows autoradiographs from control (**A**) and nodularin-treated (**B**) cells. The non-phosphorylated silver-stained SOD1 (spot N1) appeared as a white spot on the autoradiographs in both control- and nodularin-treated hepatocytes. Nodularin treatment led to increased abundance of the two phosphorylated SOD1 spots (P1 and P2). The effect was more pronounced than observed with a lower concentration of nodularin (Figure 2).

Nodularin-induced phosphorylation of SOD1 was further supported by immunoprecipitating SOD1 from total lysate of hepatocytes pre-labeled with radioactive phosphate. Again, the three forms (N1, P1 and P2) of SOD1 were recognized (Figure 4). The silver stained N1 spots in both control and nodularin-treated hepatocytes could be observed as white “spots” on the autoradiograms (Figure 4). P1 and P2 showed increased phosphate labeling after two minutes of exposure to 5 µM nodularin. The increased phosphorylation was more pronounced than in the hepatocytes treated for a longer period with a lower dose (200 nM) as seen in Figure 2.

Phosphorylation of SOD1 has previously only been shown in human erythrocytes [16], but the function of this phosphorylation is unknown. Apart from identifying phosphorylation of Ser2 [16], which is not found in rat, Wilcox *et al*. also suggested Thr58 or Ser59 to be phosphorylated. Thr58 is a highly conserved amino acid and also a possible phosphorylation site in nodularin-exposed rat hepatocytes (Figure 3). Our autoradiographs showed that nodularin-exposed hepatocytes possessed two phosphorylated SOD1 spots (Figure 2 and Figure 4), both showing an acidic shift compared to the non-phosphorylated form. The tryptic peptide of amino acid 24–69 was the only peptide that did not appear in these phosphorylated forms (P1 and P2) when compared to the non-phosphorylated form (N1) (Figure 3). This peptide contains a number of potential Ser and Thr phosphorylation sites.

### 2.2. Ca^2+^/Calmodulin-Protein Kinase II Inhibitor KN93 Delays Nodularin-Induced ROS Development and Inhibits Post-Translational Modifications of SOD1

**Figure 5:**
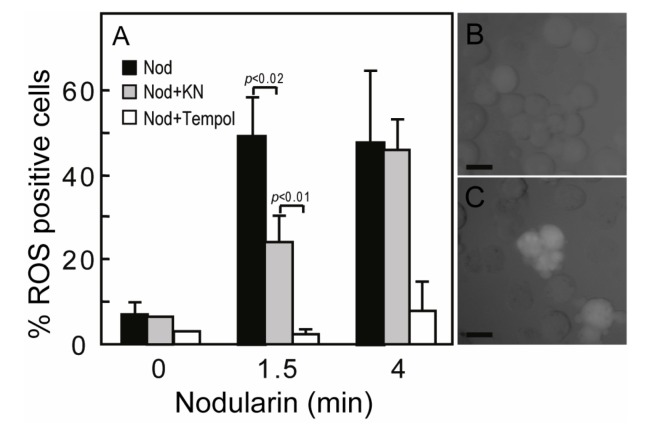
Nodularin-induced ROS development can be delayed by CaMKII inhibitor KN93. (**A**) Hepatocytes were incubated with 5,6-chloromethyl-2',7'-dichlorodihydrofluorescein (CM-H_2_DCFDA) with or without either KN93 (30 µM) or the SOD1 mimic TEMPOL (5 mM) prior to addition of nodularin (5 µM). Hepatocytes were fixed and evaluated immediately by fluorescence microscopy. Hepatocytes were scored ROS positive when fluorescence increased clearly above hepatocyte auto-fluorescence (**B**, **C**). Both KN93 and TEMPOL inhibited nodularin-induced development of ROS. Panel B shows control cells and panel C nodularin-exposed cells. Values are the mean +/− SEM of 3–5 separate experiments. *p*-values; Student’s *t*-test. Bars: 20 µm.

SOD1 is known as a broad acting scavenger of oxidative stress [12] and the observed SOD1 phosphorylation could therefore be related to increased ROS development. Microcystin has previously been shown to increase ROS in primary hepatocytes [17]. Nodularin exposure also resulted in a ROS development, both at 200 nM (data not shown) and 5 μM exposure (Figure 5A). The ROS development was efficiently inhibited by the SOD mimic TEMPOL (Figure 5A). As we have previously shown that Ca^2+^/calmodulin protein kinase II (CaMKII) is required for phosphatase inhibitor-induced apoptosis [3], we also tested whether CaMKII activity had any effect on nodularin-induced ROS development. Interestingly, KN93, a CaMKII inhibitor, did inhibit ROS development (Figure 5). This is in agreement with a study of microcystin-induced apoptosis, in which CaMKII was proposed to act upstream of mitochondrial ROS production [4].

**Figure 6:**
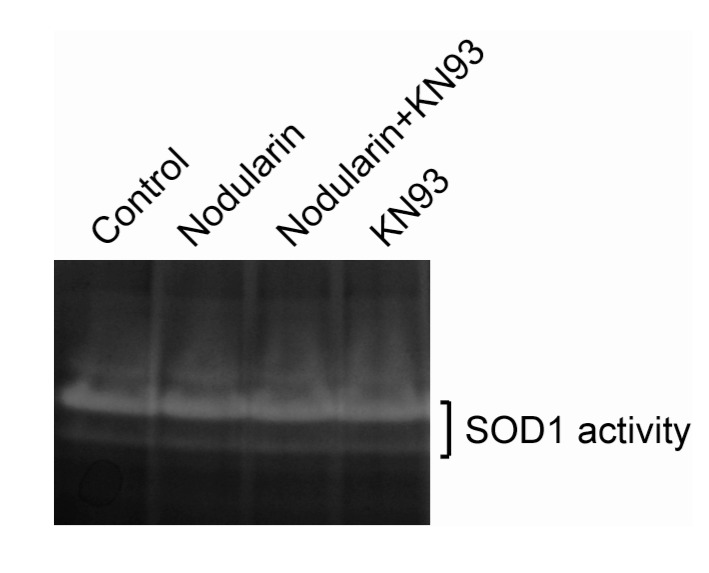
SOD1 activity is retained in nodularin-exposed hepatocytes. Hepatocytes were pre-incubated with or without CaMKII inhibitor KN93 (30 µM) prior to addition of nodularin (200 nM) or left untreated. After 10 min of incubation cells were harvested, lysed, and equal amount of proteins were separated on a native gel and stained for SOD1 activity. See Experimental Section for further details.

Next, we examined whether nodularin exposure, resulting in increased ROS (Figure 5) and phosphorylation of SOD1 (Figure 2 and Figure 4), had any effect on the enzymatic SOD1 activity. As can be seen in Figure 6, SOD1 activity appeared to be stable in the nodularin-exposed cells. Neither did SOD1 activity depend on CaMKII activity.

**Figure 7:**
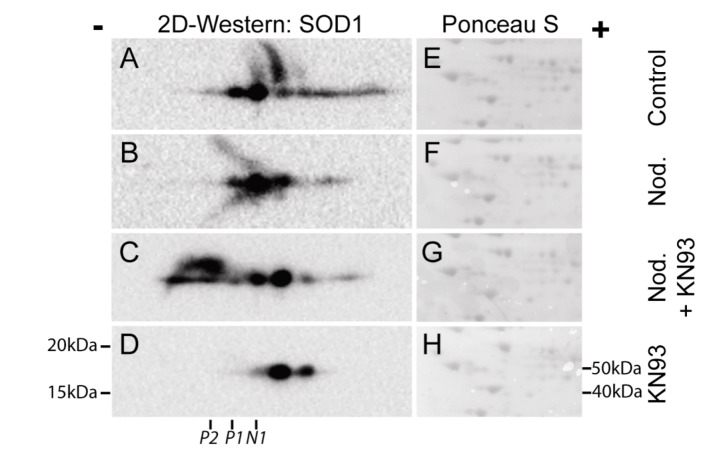
CaMKII inhbitor KN93 regulates post-translational modification of SOD1. Hepatocytes were pre-incubated without (**A**, **B**) or with 30 µM KN93 (**C**, **D**) prior to addition of nodularin (200 nM). After 10 min of exposure nodularin samples (**B**, **C**) and controls (**A**, **D**) were TCA-precipitated and proteins were separated by two-dimensional gel electrophoresis. Gels were blotted and the membranes were stained with Ponceau S (**E**–**H**) before they were probed with anti-SOD1 antibody. SOD1 spots were horizontally aligned based on the Ponceau S staining. Positions of N1, P1 and P2 (Figure 2) are indicated.

We have previously shown that the majority of microcystin-induced phosphorylation events depend on CaMKII activity [3]. Using two-dimensional gel electrophoresis, followed by Western blotting, we elucidated the role of CaMKII in post-translational modification of SOD1 (Figure 7). In non-treated hepatocytes a number of SOD1 spots were observed, both on the basic and the acid side of the major SOD1 spot (N1) (Figure 7A). Nodularin exposure resulted in an acidic shift of the most basic SOD1 spots (Figure 7B). A SOD1 spot at the position of P1 (Figure 2) was observed, but a similar positioned spot was also observed in the control cells. This indicates that at position P1 there is a mixture of phosphorylated SOD1 and SOD1 with another modification giving a similar acidic shift. Similar acidic pI shifts will be observed in cases where cysteines are modified. Fujiwara *et al.* [18] showed that Cys111 of recombinant SOD1could be converted to cysteine sulfinic acid and to cysteine sulfonic acid, giving acidic pI shifts, when exposed to H_2_O_2_. It should be noted that this cysteine modification did not alter SOD1 activity [18], which is in accordance with our observations (Figure 6). Taken into account the nodularin-induced ROS development (Figure 5), the SOD1 modified spots we observe (Figure 7) can indeed be mixtures of phosphorylated and oxidized forms of SOD1. To identify possible CaMKII-regulated oxidation or phosphorylation sites of SOD1, one would need to proceed in a transfectable cell model using site-directed mutagenesis. Unfortunately, the hepatocyte specific nodularin can then not be used as an apoptosis inducer.

### 2.3. SOD1 Concentration Is High in the Sub-Membranous Area and in the Apoptotic Buds of Nodularin-Exposed Hepatocytes

We next studied the sub-cellular localization of SOD1 in nodularin-exposed hepatocytes to possibly find any indication of the functional role of SOD1 post-translational modification.

**Figure 8:**
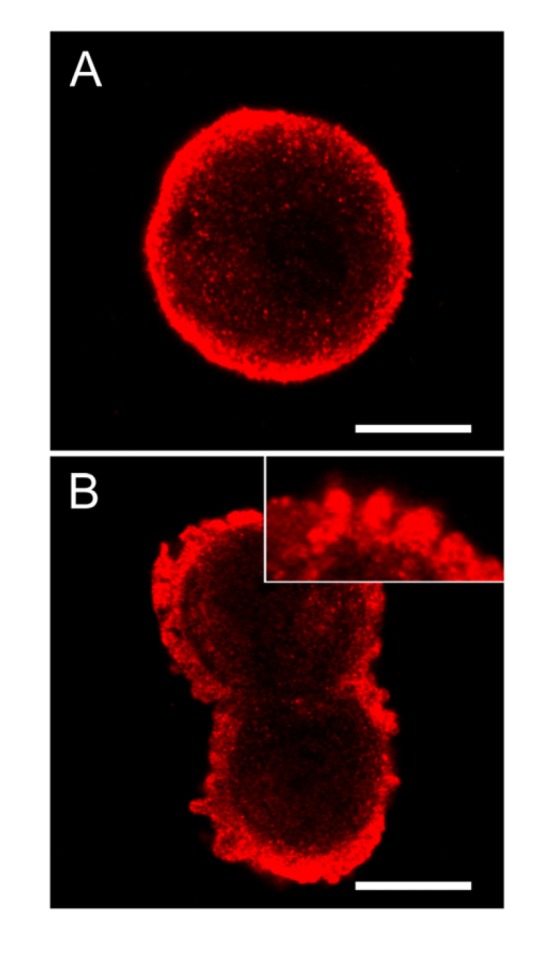
SOD1 is highly concentrated below the cellular membrane in hepatocytes and also in the apoptotic cell membrane buds. In untreated control cells, high concentration of SOD1 was found in the sub-membranous area (**A**). After 2 min of nodularin treatment (5 µM) SOD1 localized to the apoptotic cellular membrane buds and stretched into the cytosol (**B** and insert of **B**). To visualize SOD1 cells were immunostained with anti-SOD1 antibody. Bars: 10 µm.

In control cells of hepatocytes in suspension cultures, the extent of SOD1 was highly increased in the area just below the cellular membrane (Figure 8A). Following nodularin treatment, the sub-membranous SOD1 seemed to be concentrated into the apoptotic buds, leaving “tails” of SOD1 staining in the cytoplasma (Figure 8B). 

**Figure 9:**
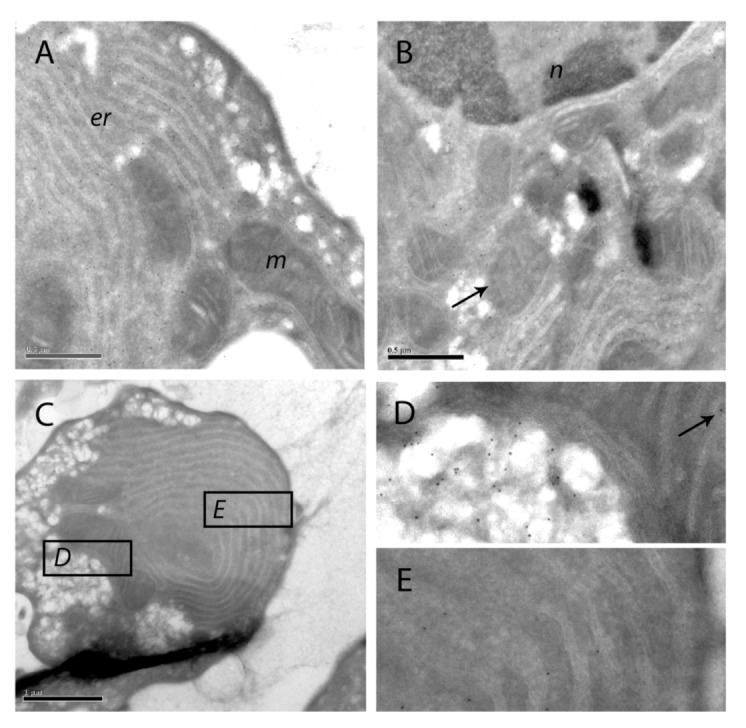
Immuno-gold labeling of SOD1 on cryosections of apoptotic hepatocytes. SOD1 was visualized with protein A-gold labeling of SOD1 in hepatocytes treated with 200 nM nodularin for 20 min. SOD1 was found throughout the cytosol and, to a minor degree, in the euchromatin area of the nucleus (n) (**A** and **B**). Minor amounts of SOD1 were observed in mitochondria (m) (arrows) and in the cisternae of endoplasmatic reticulum (er). In the apoptotic buds (**C**) a high concentration of SOD1 was found in the matrix part of the buds and between the tightly packed layers of endoplasmatic reticulum (**C**–**E**).

To study this in more detail we cryosectioned hepatocytes in suspension and labeled them with protein A-gold particles after incubation with anti-SOD1 antibody. In control cells, the electron microscopic resolution of SOD1 localization in isolated hepatocytes resembled that previously observed in hepatocytes of unexposed rat livers (data not shown) [19]. Thus, the majority of SOD1 was found in the cytoplasm, including in the microvilli, with minor amounts in the mitochondria and endoplasmatic reticulum (data not shown). In late-staged apoptosis following nodularin exposure, labeling density of SOD1 was slightly higher close to the cell surface area than around the cell nucleus (Figure 9A,B). The cisterna of the endoplasmatic reticulum (er) and the mitochondria (m) contained only minor amounts of SOD (Figure 9), similar as observed in the unexposed hepatocytes and liver (data not shown) [19]. 

The immunofluorescence-based observation of high SOD1 concentrations in the apoptotic buds was further supported by the protein A-gold-labeling. Thus, in apoptotic buds of the hepatocytes (Figure 9C) high density of labeling was found in the cytoplasmic matrix part of the buds and minor amounts in the mitochondria (Figure 9D). SOD1 was also found between the layers of tightly packed endoplasmatic reticulum in the buds (Figure 9E).

Mitochondrial SOD1 has been proposed to have a pro-apoptotic role by being released together with cytochrome C from mitochondria-dependent hepatocyte apoptosis [20]. When we investigated sub-cellular distribution of SOD1 in nodularin-induced apoptotic hepatocytes we still could observe SOD1 in mitochondria (Figure 9, arrows). We did not observe any ultrastructural damage or swelling of mitochondria, which accompanies mitochondrial-dependent apoptosis [20] (Figure 9). This suggests that mitochondrial SOD1 release does not have a major role in nodularin-induced apoptosis. On the other hand, the highly concentrated appearance of SOD1 in the sub-membranous area and in the apoptotic buds would make it possible for SOD1 to act in the dramatic reorganization of the cytoskeleton that occurs during the nodularin-induced apoptotic budding (Figure 1) [10,11].

### 2.4. SOD1 Co-localization with Actin Filaments Is Disrupted in Hepatocytes Exposed to Nodularin

**Figure 10:**
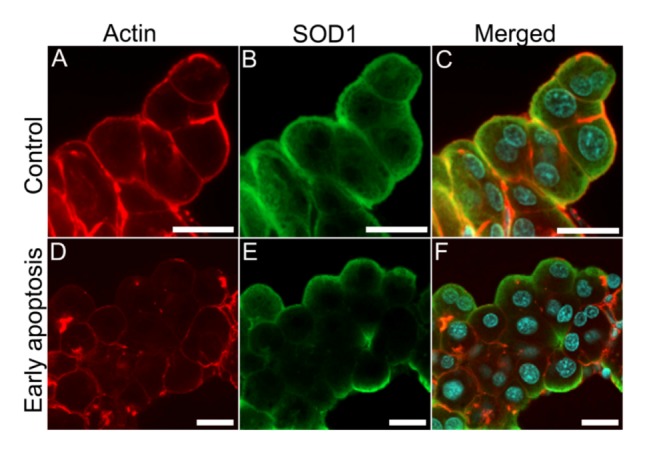
SOD1 and actin co-localization is disrupted in nodularin-exposed hepatocytes. In unexposed control hepatocytes, a high degree of both SOD1 and actin fibers were found co-localized in the sub-membranous area (yellow) (**A**–**C**). After exposure with 200 nM nodularin for 10 min the actin cytoskeleton reorganized into aggregates while SOD1 was still found in the sub-membranous area (**D**–**F**). Cells were fixed and labeled with anti-SOD1 antibody (green), followed by incubation with rhodamin-conjugated phalloidin (red), and DAPI (blue) for nuclear staining. Bars, 20 µm.

Nodularin-induced apoptosis involves reorganization of the cell cytoskeleton, including actin filaments (Figure 1D,E), in order to develop apoptotic morphology (Figure 1). Interestingly, expression of SOD1 has been shown to be essential for the preservation of the cytoskeleton [15], and disease-related SOD1 mutants interact directly with the actin cytoskeleton [14,21]. We therefore compared the localization of SOD1 and actin filaments in nodularin-exposed hepatocytes. In control cells SOD1 and actin showed an overlap in the sub-membranous area (Figure 10A–C). In the early stage of nodularin-induced apoptosis, before budding started to appear, this co-localization was partially lost (Figure 10D–F).

**Figure 11:**
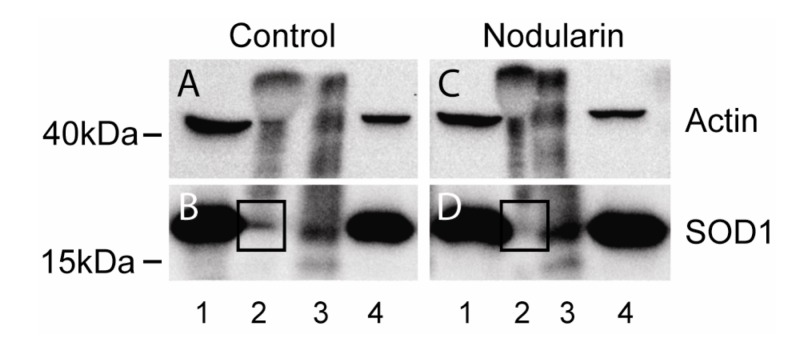
Co-immunoprecipitation of SOD1 and actin. Hepatocytes were left untreated (**A**, **B**) or exposed to 200 nM nodularin for 20 min (**C**, **D**). Total cell lysates were immunoprecipitated with anti-actin antibody and eluted proteins were separated by SDS-PAGE followed by Western blotting usinganti-actin and anti-SOD1 antibodies. Lane 1: total lysate, 2: glycine eluate, 3: SDS-eluate, and 4: unbound. Immunoprecipitated SOD1 is indicated in boxes.

We next tested whether the SOD1-actin co-localization (Figure 10) could be shown to be a direct or indirect binding between the two proteins using co-immunoprecipitation. Both proteins are high abundant proteins which always increase the risk of unspecific binding [22] and immunoprecipitation of F-actin is usually not very efficient. It should be noted that previous studies have only been able to immunoprecipitate minor amounts of wild type SOD1 together with actin [14]. Only a minor amount of SOD1 was pulled down together with actin also in our untreated cells (Figure 11A,B). On the other hand, in the nodularin-treated cells we did not observe any SOD1-actin co-interaction (Figure 11C,D). This indicated that the observed co-localization of SOD1 and F-actin (Figure 10) may involve co-interaction between the two proteins.

### 2.5. SOD1 May Operate in Cellular Micro-Domains to Control Cytoskeletal Reorganization

Apart from being known as a scavenger of oxidative stress [12], SOD1 has more recently also been shown as a redox regulatory protein at discrete sub-cellular domains [13]. In these sub-cellular domains, ROS-sensitive kinases such as mitogen-activated protein kinase (MAPK) p38 and CaMKII are suggested to have key roles [23,24]. Since only a minor amount of the total SOD1 was shown to be phosphorylated (Figure 2 and Figure 4) this suggests that SOD1 phosphorylation may have a discrete regulatory function and not necessarily be a way to protect the cells from the major mitochondrial-linked ROS burst that occurs in nodularin-exposed hepatocytes [17,25]. 

Reorganization of actin filaments seems to depend on locally produced ROS [26,27]. Rac1, an activator of NADPH oxidase, is a mediator of this redox-dependent reorganization of the actin cytoskeleton [26]. Interestingly, SOD1 has been shown to interact with Rac1 in a redox-dependent manner [13]. This interaction keeps Rac1 in an active state, making it possible for Rac1 to participate in actin reorganization [26,28]. Phosphorylation and possibly also oxidation of SOD1 may regulate its ability to interact with other proteins.

Phosphorylation of SOD1 (Figure 2 and Figure 4) is accompanied with CaMKII-dependent phosphorylation of myosin light chain (MLC) (data not shown) in nodularin-exposed hepatocytes. Phosphorylated MLC has been shown to be a target for activated Rac1 [29] and has also proven to be necessary for actin-myosin-based apoptotic budding [30]. Nodularin-induced phosphorylation of MLC and SOD1 may therefore be involved in regulating that apoptosis-related cytoskeletal reorganization and budding in nodularin-exposed hepatocytes.

## 3. Experimental Section

### 3.1. Materials

Nodularin was purified from cyanobacteria (Nodularia) as described in [31], and was a generous gift from Herfindal, L. and Døskeland, S.O. University of Bergen. KN93 was purchased from Calbiochem [^32^P]orthophosphate (10 mCi/mL), IPG-buffer, linear immobilized pH 5.3–6.5 gradients, CHAPS and Coomassie Brilliant Blue R250 were from GE Healthcare. The matrix 2,5-dihydroxybenzoic acid was purchased from Bruker Daltonics (Bremen). Sequencing grade modified trypsin was obtained from Promega. Rhodamin-conjugated phalloidin and all other biochemicals were purchased from Sigma-Aldrich.

### 3.2. Cells

Hepatocytes were isolated from male Wistar rats (120–200 g) by *in vitro* collagenase perfusion and kept in suspension cultures as earlier described [1]. In some experiments cryopreserved hepatocytes were used (Celsis *in Vitro* Technologies). Cells were incubated in capped vial in pre-gassed low-phosphate (0.1mM) Krebs–Ringer bicarbonate buffer (10 mM Hepes (pH 7.4), 120 mM NaCl, 5.3 mM KCl, 0.01 mM KH_2_PO_4_, 1.2 mM MgSO_4_, 1.0 mM CaCl_2_) with 5 mM lactate, 5 mM pyruvate, and 0.5% bovine serum albumin.

### 3.3. Evaluation of Apoptosis and ROS Detection

Apoptosis was evaluated in an invert phase microscope by observing the morphology of cells fixed in 2%–4% formaldehyde in PBS (pH 7.4). Apoptotic cells were easily discriminated from normal and necrotic cells by the appearance of cell surface buds. For photographs of hepatocytes, a small volume of fixed cell suspension was put on a glass slide and developed on a Zeiss Axiophot microscope equipped with differential interference contrast.

For measurement of intracellular ROS, hepatocytes were incubated in the dark with 50 μg/mL 5,6-chloromethyl-2',7'-dichlorodihydrofluorescein diacetate (CM-H_2_DCFDA, Molecular Probes) at 37 ºC for 30 min prior to addition of nodularin. Cells were fixed and analyzed immediately using fluorescence microscopy.

### 3.4. Labeling of Cellular Phosphoproteins and Sample Preparation

Suspension cultures of rat hepatocytes (1.7 mill cells per mL) were pre-incubated with ^32^Pi (1000 µCi/mL) for 35 min before addition of nodularin. After incubation with or without nodularin (10 min with 200 nM or 2 min with 5 µM) cells were spun down and the pellet was snap frozen in liquid nitrogen.

In our initial experiments and attempt to identify phosphoproteins, frozen pellets from 3 mL suspension cultures were resuspended in 10 mM Na-Hepes pH 7.00, 50 mM MgCl, 15 mM KCl with protease inhibitor cocktail (cOmplete, Roche). An equal amount of 0.5 mM sucrose was added and nuclei were removed by centrifugation at 500*g* for 10 min. Supernatant was added EDTA (final concentration 10 mM) and centrifuged at 16,000*g* for 10 min. All the steps above were performed at 4 °C. Supernatant (mainly consisting of cytosolic proteins) was precipitated with acetone over night at −20 °C. Samples were spun 20 min at 16,000*g* and pellets were resuspended in 100 µL 2-D sample buffer (9.8 M urea, 100 mM DTE (1,4-dithioerythritol), 1% *v*/*v* IPG-buffer 4–7, 4% *w*/*v* CHAPS and 0.2% *w*/*v* SDS).

Frozen pellets used for immuno-precipitation of SOD1 were resuspended in RIPA lysis buffer (50 mM Tris pH 7.4, 150 mM NaCl, 1% NP-40, 0.25% sodiumdeoxycholate, 1 mM EDTA) with protease inhibitor cocktail, 50 mM NaF, 5 mM Sodium Pyrophospate and 1 mM Na_3_VO_4_. Possible unspecific binding was removed by incubating cell lysate for 1 h with sepharose protein A and similarly with sepharose G. Anti-SOD1 antibody (10 µg/sample) was added to the supernatant and incubated over night on a rotary wheel. All steps were performed at 4 °C. The next day, sepharose G beads were added to the suspension and left for two hours. Beads were washed twice in 1:10 RIPA buffer and proteins were eluated with 2-D sample buffer.

### 3.5. Two-Dimensional Gel Electrophoresis

Samples for mass spectrometry analysis were cup-loaded onto rehydrated 13 cm strips (pH 5.3–6.5) and run on an IPGphor (GE Healthcare). Isoelectric focusing (IEF) parameters were as follows: (i) 30 V for 12 h; (ii) 100–500 V in 3 h; (iii) 500 V for 3 h; (iv) 500–1000 V in 3 h; (v) 1000 V for 3 h; (vi) 1000–8000 V in 3 h; (vii) 8000 V for 20 h, with a 50 µA per strip maximum at 20 °C. Strips were subjected to second dimensional separation using 12% SDS-PAGE. Separated proteins were visualized using Coomassie Blue, Sypro Ruby or silver staining.

Samples for 2D-Western were precipitated with trichloroacetic acid (TCA) (final 10%), washed in 5% TCA followed by acetone, and resuspended in 2D-sample buffer. 

Immunoprecipitates were analyzed by using the ZOOM^®^ IPGRunnerTM Mini-Cell System (Invitrogen) using IPG-strips pH 4–7. Manufactures protocol was followed except that the IEF parameters were: (i) 200 V for 30 min; (ii) 450 V for 15 min; (iii) 750 V for 20 min; and, (iiii) 2000 V for 60 min. 

Gels were dried onto filter paper and exposed to autoradiography films at −80 ºC with intensifying screens. For protein identification, the autoradiographs were printed on transparencies and these were used to cut out phosphorylated proteins from the Coomassie-stained gels.

For immunoblotting, gels were transferred to PVDF membranes, blocked with 5% non-fat dry milk and probed with with anti-SOD1 antibody (Stressgen #SOD1-100) or anti-actin antibody (Sigma #2228) followed by a HRP-conjugated secondary antibody, the protein bands were detected by chemiluminescence (Supersignal, Pierce).

### 3.6. Digestion of Proteins

Spots cut out from dried gels were re-swelled in deionized water and filter paper backing was removed. Spots were cut into smaller pieces, washed twice for 15 min in 1 mL acetonitrile/water (1:1) and dehydrated by using 100% acetonitrile (HPLC-grade). In-gel digestion were then performed o/n at 37 °C with 20 µL 2.5 µg/mL trypsin in 50 mM ammonium bicarbonate buffer, pH 8. The digestion was terminated by adding 3 µL 5% TFA and centrifuged at 16,000 × *g* for 15 min. The supernatant was collected, and peptides left in the gel pieces were extracted by adding 20 µL 60% acetonitrile in 0.1% TFA and incubated at room temperature for 15 min. At the end of incubation, the gel pieces were centrifuged one more time for 15 min at 16,000 × *g*, and supernatants were collected and mixed with the previous ones. The collected supernatants from each sample were dried on a speedvac to a final volume of approx 5 µL.

### 3.7. Up-Concentration, Desalting and Elution of Peptides

Prior to analysis using MALDI TOF mass spectrometry, a nano-column made from Eppendorf GELoader tips (Eppendorf) containing oligo-R3 reversed phase material, was used to up-concentrate peptides and to remove salts and other contaminants. Peptides absorbed to the oligo-R3 resin, were eluted directly on the MALDI-target in small drops by 1 µL 10 mg/mL 2,5-dihydroxybenzoic acid in 50% acetonitrile and 1% phosphoric acid and air-dried immediately prior to analysis.

### 3.8. Mass Spectrometry

Positive ion MALDI TOF mass spectra were acquired using an Ultraflex MALDI Tof/Tof (Bruker Daltonics) mass spectrometer, operated in reflectron mode. After time-delayed extraction, the ions were accelerated to 25 kV for MALDI-TOF mass spectrometric analysis. A total of 500 laser shots were acquired and signal averaged per mass spectrum. After calibration and peak detection, the peptide mapping data were compared to the Swiss-Prot database and later confirmed by database search using Mascot 2.2.1 and database comparison with human entries (18 1117 sequences) from UniProt database [32] Peptides considered as candidates for MS/MS-sequencing, was selected as precursor ion for additional analysis using positive ion MALDI TOF mass spectrometry with post-source decay (PSD). Precursor ions for PSD analysis were selected using an ion gate at a resolving power of ~500. 

### 3.9. SOD1 Activity Assay

Cells were harvested by centrifugation (1000*g*, 1 min, 4 °C) and washed twice with ice-cold PBS, pH 7.4. Cells were resuspended in 150 µL lysis buffer (50 mM potassium phosphate, pH 7.8 with protease inhibitor cocktail (Roche)) and lysed by physical shearing using a 23 G needle. After centrifugation (15,000*g*, 15 min, 4 °C) to remove cellular debris, equal amounts (38 μg) of proteins were loaded on a precast 4%–16% Bis-Tris NativePAGE Novex gel (Invitrogen) and electrophoresis was performed according to manufacturer’s manual. SOD activity assay staining was performed as described by Beauchamp and Fridovich [33]. The gel was incubated in SOD activity assay-solution (10 mg NBT, 7.5 mg riboflavin and 75 µL TEMED in 75 mL of 50 mM potassium phosphate buffer, pH 7.8) at RT for 30 min in the dark, before it was rinsed twice with water and then exposed to light until the SOD bands appeared as white band on a dark blue background. 

### 3.10. Immunofluoresence and Actin Labeling

Hepatocytes were fixed in 4% para-formaldehyde, permeabilized with 0.1% Triton X-100, and stained in blocking solution with anti-SOD1 antibody (#SOD-101 Stressgen) at 1:1000 followed by Cy3- or FITC-conjugated secondary antibodies (Jackson ImmunoResearch Laboratories). For visualization of actin the permeabilized cells were incubated with rhodamin-conjugated phalloidin. Cells were imaged with a Zeiss LSM 510 confocal microscope.

### 3.11. Cryosectioning and Gold Labeling

Suspensions cultures of hepatocytes were fixed in 4% paraformaldehyde, 0.2 M PIPES (pH 7.2) and 0.1% glutaraldehyde. Pellets were washed in PBS (3×) and covered in 10% pig skin gelatin in PBS (pre-warmed at 37 °C) and incubated for 25 min at 37 °C. Samples were placed on ice for the gelatin to set and then pellets were cut into smaller blocks. Blocks were covered with PVP-2.3 M sucrose and kept at 4 °C overnight and thereafter placed on specimen stubs and frozen in liquid nitrogen. Samples were crysectioned and mounted on specimen grids. Grids were floated on drops of 0.1 M ammonium chloride in PBS for 10 min then washed in PBS and blocked in 0.5% fish skin gelatin in PBS for 10 min. Grids were incubated with anti-SOD1 antibody (1:50) for 30 min, washed (3×) in PBS and incubated with protein A-gold (1:30) for 20 min. Sections were stained methyl cellulose/uranyl acetate and viewed in a Jeol JEM-1230 transmission electron microscope.

### 3.12. Immunoprecipitation of Actin

All steps were carried out at 4 °C. Cells were harvested by centrifugation, washed twice with PBS, and lysed in lysis buffer (150 mM NaCl; 50 mM Tris-HCl, pH 7.4; 1 mM EDTA; 1% Triton X-100; 50 mM NaF; 5 mM Na_4_P_2_O_7_; 1 mM Na_3_VO_4_; protease inhibitor cocktail (Roche)) by incubation for 15 min. Cell lysate was cleared and equal amounts of proteins from untreated and nodularin-exposed cells were incubated on a rotating wheel for 1 h with rabbit IgG to remove unspecific binding. 50 μL Protein A sepharose beads were washed twice with TBS pH 7.4 and added to the lysate/rabbit IgG mixture and incubated overnight. Next day, the mixture was centrifuged (1000*g*, 2 min). 50 µL new Protein A sepharose beads (washed twice with TBS pH 7.4) were added to the lysate and incubated 1 h to remove all IgG. The lysate was incubated 2 h with actin antibody (rabbit polyclonal, Sigma, #A5060) for 2 h. 50 µL Protein A sepharose beads were washed twice with TBS pH 7.4 and then added to the lysate/anti-actin antibody mixture and incubated over night. The next day, the mixture was centrifuged to separate the supernatant (unbound fraction) from the beads. The beads were washed 5× with TBS pH 7.4, then briefly washed with 0.1 M glycine pH 2.5, before incubation with glycine for 1 h at 4 °C to elute the bound proteins. Both glycine fractions were pooled. After washing the beads twice in TBS pH 7.4, the beads were boiled in 2× SDS loading buffer for 5 min, to release any remaining proteins. 

## 4. Conclusions

We show that the Cu,Zn superoxide dismutase (SOD1) is rapidly phosphorylated and possibly also oxidized in hepatocytes exposed to the cyanobacterial protein phosphatase inhibiting toxin nodularin. These post-translational modifications do not alter the enzymatic SOD1 activity. In unexposed hepatocytes SOD1 co-localizes with the cortical actin cytoskeleton. After exposure to nodularin this co-localization is disrupted and SOD1 is highly concentrated in the appearing apoptotic buds. Our results suggest that SOD1 and SOD1 phosphorylation may play a regulatory role in the dramatic actin reorganization induced by nodularin.
